# The dominance of dorsal scapular artery as the blood supply to muscles of the back in the absence of two primary vessels: a cadaveric case report

**DOI:** 10.1007/s00276-024-03376-z

**Published:** 2024-05-08

**Authors:** Janay Rocha, Robert Chalk, Arunabh Bhattacharya

**Affiliations:** 1 University of the Incarnate Word School of Osteopathic Medicine, 7615 Kennedy Hill Drive, San Antonio, TX USA; 2 Department of Applied Biomedical Sciences, University of the Incarnate Word School of Osteopathic Medicine, 7615 Kennedy Hill Drive, San Antonio, TX 78235 USA

**Keywords:** Thyrocervical trunk, Suprascapular artery, Transverse cervical artery, Dorsal scapular artery

## Abstract

**Purpose:**

Understanding of rare or unknown anatomical variations of the vasculature of the neck is critical to reduce the risk of complications during surgeries and other invasive procedures in the neck and shoulder regions.

**Methods:**

Bilateral dissection of the neck and muscles of the back of an 87-year-old Caucasian male donor was performed to demonstrate the origin, course and termination of the arteries that arise in the neck.

**Results:**

Several anatomical variations were noted on the right side of the neck of the donor body – (i) only inferior thyroid and ascending cervical arteries originated from the thyrocervical trunk (TCT), from the first part of the subclavian artery (SA), whereas the transverse cervical (TCA) and suprascapular (SSA) arteries were entirely absent, (ii) Dorsal scapular artery (DSA) emerged normally from the third part of the SA. However, after supplying the rhomboids and levator scapulae muscles, DSA provided two additional branches to the trapezius muscle and a branch to the supraspinatus muscle. Interestingly, the branches to the trapezius muscle from the DSA were the only sources of blood supply to the muscle.

**Conclusion:**

We report a unique anatomical variation involving the absence of the TCA and SSA from the TCT. The unilateral absence of these major vessels and the branches of DSA supplying the trapezius and supraspinatus muscles have not been reported previously in the literature in a single case report. This case study may provide useful information for head and neck reconstruction and shoulder repair surgeries.

## Introduction

The thyrocervical trunk (TCT) arises superiorly from the first part of the subclavian artery (SA), medial to the anterior scalene muscle (ASM), and provides four main arterial branches: the inferior thyroid artery (ITA), ascending cervical artery (ACA), suprascapular artery (SSA), and transverse cervical artery (TCA) [1, 2]. The SSA and TCA supply two of the rotator cuff muscles and trapezius muscle, respectively, while the ACA and ITA supply structures of the anterior neck including muscles and endocrine organs [2]. The dorsal scapular artery (DSA), a branch of the third part of the SA, provides blood supply of the levator scapulae and rhomboid muscles, and anastomosis around the scapula [2]. Thus, DSA, together with the SSA and TCA are important arterial contributors to the muscles of the back and shoulder.

Studies involving cadaveric neck dissections, computed tomography angiographies (CTA), and case reports have demonstrated variations in the branching of the TCT [1, 3–7]. Studies have also reported anatomical variations of the SSA [8–11], TCA [5, 12] and DSA [13, 14]. However, the literature on the absence of the SSA and TCA is limited [5]. Knowledge of anatomical variations in the arterial supply of the neck and muscles of the back can reduce surgical error and intraoperative complications [15–18]. In this case report, we describe the unilateral absence of the SSA and TCA from the TCT, and branches from the DSA providing blood supply to the trapezius and supraspinatus muscle on a male donor body.

## Case report

**Fig. 1:**
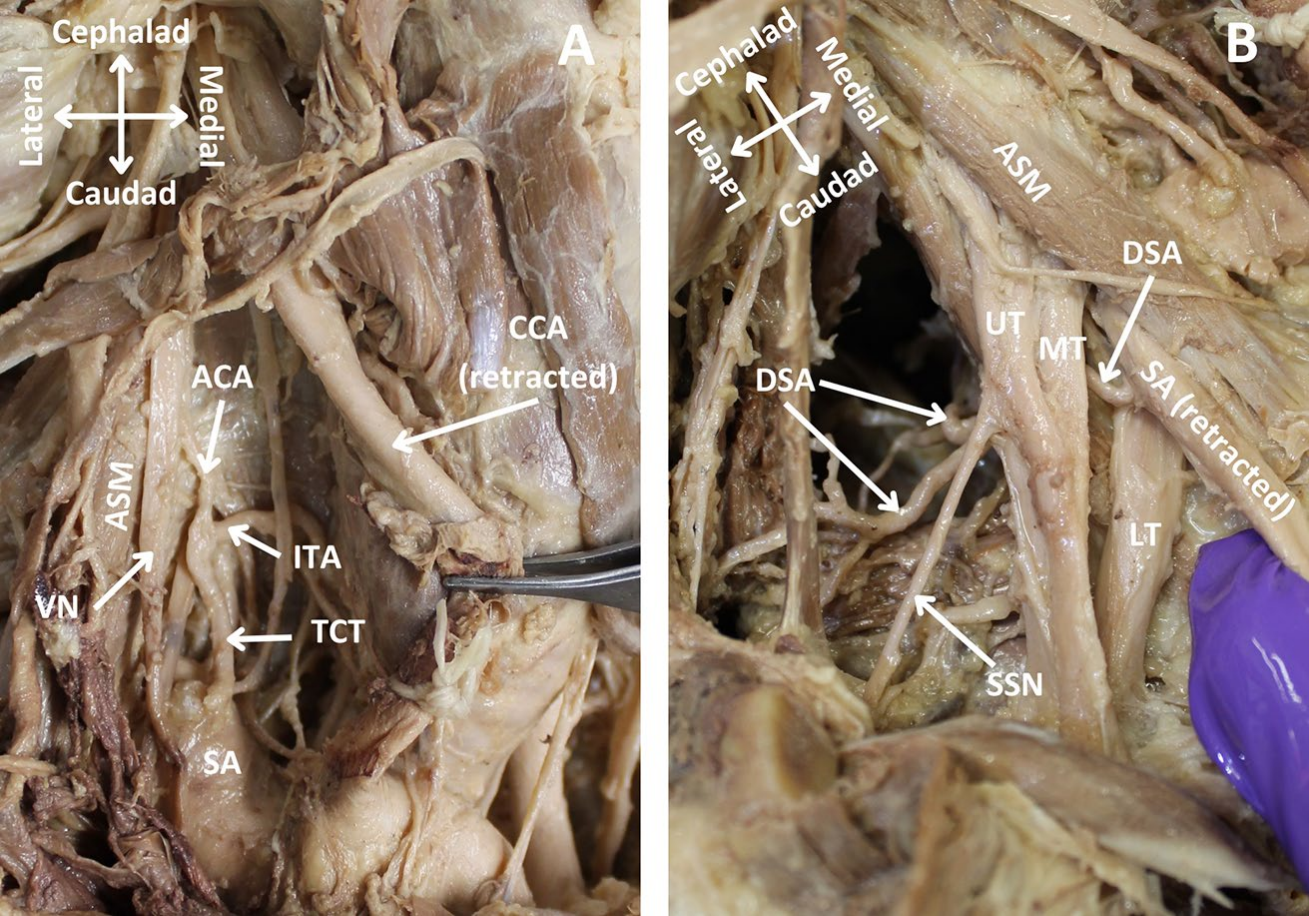
Anterior view of the right side of the neck. The right side of the neck was dissected to trace the origin, course and termination of the arteries of the neck that arise from the subclavian artery. (**A**) Only inferior thyroid and ascending cervical arteries arose from the thyrocervical trunk, whereas transverse cervical and suprascapular arteries were absent. (**B**) Dorsal scapular artery arose normally from the third part of subclavian artery and gave rise to multiple branches. CCA: common carotid artery, SA: subclavian artery, TCA: transverse cervical artery, SSA: suprascapular artery, ITA: inferior thyroid artery, TCT: thyrocervical trunk, SSN: suprascapular nerve, VN: vagus nerve, ASM: anterior scalene muscle, UT: upper trunk of brachial plexus, MT: middle trunk of brachial plexus, LT: lower trunk of brachial plexus

**Fig. 2:**
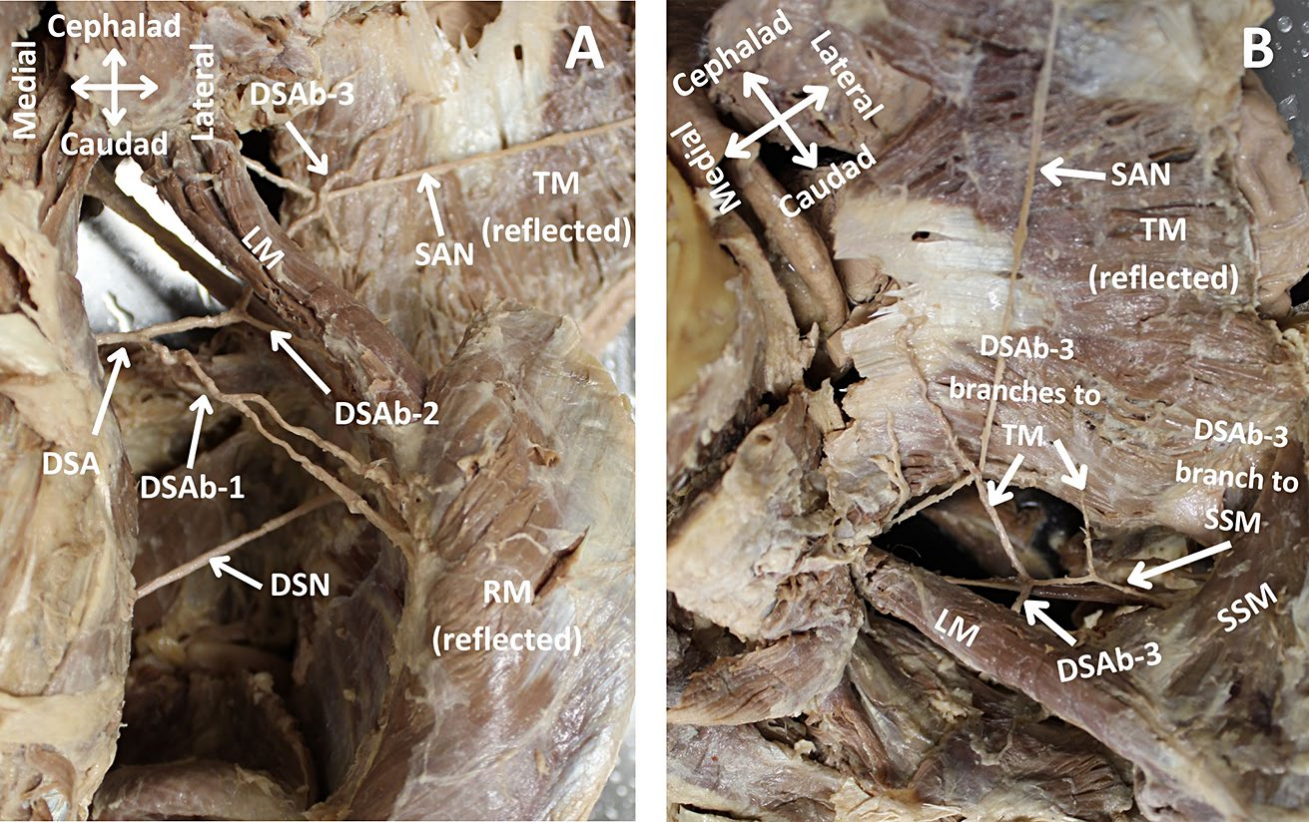
Posterior view of the musculature and vasculature of the back. The superficial muscles of the back (trapezius, levator scapulae, rhomboids) were dissected on the right side to trace the origin and course of the dorsal scapular artery. (**A**) Posterior view of the branching of the dorsal scapular artery. (**B**) Trapezius and supraspinatus muscles supplied by branches of the dorsal scapular artery. DSA: dorsal scapular artery, DSAb-1,2 and 3: first, second and third branch of dorsal scapular artery, TM: trapezius muscle, SSM: supraspinatus muscle, RM: rhomboid major and minor muscles, LSM: levator scapulae muscle, SAN: spinal accessory nerve, DSN: dorsal scapular nerve

During a routine bilateral dissection to demonstrate the neurovasculature of the neck and muscles of the back, two vessels from the TCT were noted to be absent on the right side of a donor body (race: Caucasian, age: 87, gender: male) that was donated to the Willed Body Program at the University of Texas Health, San Antonio for the purpose of research and medical education. The TCT on the right side of the donor’s neck was found to be missing the SSA and TCA, giving rise to only the ACA and ITA (Fig. 1A). The typical courses for the absent SSA and TCA were followed to their musculature: the supraspinatus, rhomboids, levator scapulae, and trapezius. While dissecting the musculature to view their blood supply, the DSA, which arose normally from the third part of the SA (Fig. 1B), was found to be the sole source of blood supply for the trapezius muscle (Fig. 2A). The DSA provided a branch to the supraspinatus muscle, in addition to its normal blood supply to the levator scapulae and rhomboid muscles (Fig. 2B). The left side of the donor’s neck was found to have a normal TCT with its four typical branches and the DSA arising from the third part of the SA.

## Discussion

Embryologically, the SA develops from the dorsal aspect of the aorta during the seventh week of development. Similarly, the ITA, ACA, TCA, and SSA are joined together during development to form the TCT [1]. While the right SA develops from the fourth aortic arch, right dorsal aorta, and right seventh intersegmental artery, the left SA arises from the left seventh intersegmental artery [19].

The TCT, arising from the SA, typically gives rise to four branches: ITA, ACA, SSA, and TCA; however, variations of these branches do exist [1]. Ostrowski et al. demonstrated that the most common branches of the TCT are the ITA, SSA, and TCA with a prevalence of 89.02% (73/82 sides of necks on CTA) and reported that the ACA branching from the TCT was rare with a prevalence of 1.22% (1/82 sides of necks on CTA) [4]. Additionally, Lippert and Pabst described that branches from the TCT, such as the ITA, SSA, and TCA may arise independently in about 5% of cases [3]. In our case, the TCT on the right side of the body gave rise to only the ITA and ACA. Zangpo et al. described a similar case using CTA where only the ITA and ACA arose from the TCT [7]. Similarly, Takafuji et al. demonstrated that a TCT with only an ACA and ITA was present in 11.1% of cases (16/144 dissected subclavian arteries) [5].

The SSA, after branching from the TCT travels inferior and lateral across the anterior scalene muscle, and SA, continuing posteriorly to the clavicle and scapula to reach the supraspinous and infraspinous fossa where it provides blood supply to the supraspinatus and infraspinatus muscles, and skin and joints around the shoulder [1, 2, 8, 10]. Variations of the SSA’s origin have been previously described in literature as the third part of the SA [10], axillary artery [11], subscapular artery [8], and the dorsal scapular artery [9]. Weiglein et al. dissected 498 sides of the neck and found the SSA to originate from the TCT (27%), SA (12%), internal thoracic artery (11%), or from trunks such as the cervico-scapular trunk (22%), dorso-scapular trunk (4%), and the cervico-dorsoscapular trunk (also known as the TCA, 24%) [6]. Pyrgakis et al. showed that the SSA originated from the third part of the SA in 1.6% (1/62 sides of the neck) of cadaveric specimens studied [10]. Takafuji et al. demonstrated that the SSA branched independently from the SA in 26% of cases (37/144 sides of the neck) and in 2.8% of cases (4/144 sides of the neck) it was completely absent [5]. In our case, the SSA did not arise from the TCT, SA, axillary artery, subscapular artery, or the DSA. The complete absence of SSA is a rare and important finding in our study.

The TCA, also known as, the transverse colli artery, is typically a branch of the TCT and travels superiorly to the SSA through the posterior triangle of the neck transversely bypassing the inferior belly of the omohyoid muscle towards the anterior portion of the trapezius muscle where it provides its blood supply [1, 2, 15, 20]. Studies previously published have demonstrated that the TCA is consistently present in 100% of cadaveric specimens and originates from the TCT [20, 21]. However, variations of the TCA exist. Takafjui et al. found that the TCA branched from the TCT in 93 out of 144 dissected sides of the neck (64.6%) [5]. In the same study, the TCA was shown to branch from the second part of the SA in 4.9% of cases and the third part of the SA in 8.3% of cases [5]. Takafjui et al. further demonstrated that the TCA was absent in 15.3% of cases (22/144 sides of the neck) and in another 22 cases the TCA branched independently from the TCT and SA (15.3%) [5]. Another study by Kumar et al. reported a bilateral absence of TCA on a single cadaveric specimen [12]. In our case, the TCA was absent on the right side of the neck from the TCT and from the SA completely without any independent branches from these vessels providing blood supply to the trapezius muscle.

The DSA has been reported to have variations in its origin [13, 14]. Lai et al. described the DSA originating from the TCA in 38 out of 79 neck dissections (48%), the second part of the subclavian artery (22%), the third part of the subclavian artery (25%), and in 5% of cases arising from the axillary artery [13]. In a cadaveric study, Verenna et al. reported that in 71% of the cases (112/158 sides of the neck), the DSA originates from the SA and in 35% (55/158) the DSA originates from the TCT [14]. In our case, the DSA originated from the third part of the SA and provided two branches that were the sole source of blood supply to the trapezius muscle, in addition to its normal blood supply to levator scapular and rhomboid muscles. Published literature shows that SSA can originate from the DSA [9], however, in our case, the SSA was completely absent. When dissecting the branch from the DSA to the supraspinatus muscle, it was evident that the vessel was not the SSA since it did not follow the typical course posteriorly to the clavicle and the supraspinous fossa [2]. A limitation of this case report was the inability to dissect the infraspinous fossa to confirm if the branch of the DSA supplying the supraspinatus muscle anastomosed with circumflex scapular artery. Literature of the DSA as the only source of blood supply for the trapezius muscles and branches to the supraspinatus muscle in a single study has not been previously reported in the literature, to the best of our knowledge.

This case report presents anatomical anomalies that are clinically significant in reconstructive procedures of the head, neck, shoulder, chest and upper back. The TCA and SSA may be used in reconstructive arterial flaps for cervicomental scars and defects of the head and neck following surgical oncology procedures. The supraclavicular flap, a fasciocutaneous flap from the skin of the shoulder and supraclavicular region, most commonly uses the TCA (93%) and SSA (7%) as primary vessels for the procedure [16]. The use of the TCA as an arterial flap can be spread to cover larger areas such as the anterior chest and clavicle [15]. Muppireddy et al. described how the TCA is a safe vessel for arterial flaps because it can anastomose and is an easily dissectible artery due to its location [18]. Additionally, patients who undergo targeted radiation therapy or treatment for recurrent tumors of the head and neck are at increased risk of damage to the vessels of the neck, specifically the external carotid artery and its branches, therefore, utilization of the TCA is a safe option for reconstructive surgery [20]. The DSA is also a clinically significant vessel used in dorsal scapular artery perforator flaps. This type of reconstructive procedure spares the use of the trapezius muscle as a flap, decreasing post-surgical complications such as donor site morbidity and loss of range of motion of the muscle [17].

## Conclusion

Variations of the TCT have been identified in previous literature, however, this case study demonstrates the rare unilateral absence of two arteries (SSA and TCA) in a single donor. Additionally, we report a unique anatomical variation where the DSA dominates as the primary blood supply to the trapezius muscle in the absence of the TCA from the TCT. This study provides knowledge of anatomical anomalies that may be useful for the education of medical students and surgeons performing reconstructive surgeries of the head and neck and procedures of the shoulder. Further directions include conducting comprehensive angiography studies in populations to assess the occurrence of these anatomical variations in vasculature of the neck and muscles of the back.

## Data Availability

No datasets were generated or analysed during the current study.
